# Effects of Fertilizer Application on Growth and Stoichiometric Characteristics of Nitrogen, Phosphorus, and Potassium in Balsa Tree (*Ochroma lagopus*) Plantations at Different Slope Positions

**DOI:** 10.3390/plants14142221

**Published:** 2025-07-18

**Authors:** Jialan Chen, Weisong Zhu, Yuanxi Liu, Gang Chen, Juncheng Han, Wenhao Zhang, Junwen Wu

**Affiliations:** Yunnan Provincial Key Laboratory for Conservation and Utilization of In-Forest Resource, College of Forestry, Southwest Forestry University, Kunming 650224, China; chenjialan@swfu.edu.cn (J.C.); my538076@swfu.edu.cn (W.Z.); lyx1997@swfu.edu.cn (Y.L.); 15581475153@163.com (G.C.); 18988443741@163.com (J.H.); 18669113324@163.com (W.Z.)

**Keywords:** *Ochroma lagopus* (balsa tree), fertilization, N-P-K stoichiometric characteristics

## Abstract

*Ochroma lagopus*, a fast-growing tropical tree species, faces fertilization challenges due to slope heterogeneity in plantations. This study examined 3-year-old *Ochroma lagopus* at upper and lower slope positions under five treatments: CK (no fertilizer), F1 (600 g/plant), F2 (800 g/plant), F3 (1000 g/plant), and F4 (1200 g/plant) of secondary macronutrient water-soluble fertilizer. Growth parameters and N-P-K stoichiometry were analyzed. Key results: (1) Height increased continuously with fertilizer dosage at both slopes, while DBH peaked and then declined. (2) At upper slopes (nutrient-poor soil), fertilization elevated leaf P but reduced branch N/K and increased root P/K. At lower slopes (nutrient-rich soil), late-stage leaf N increased significantly, with roots accumulating P/K via a “storage strategy”. Stoichiometric thresholds indicated N-K co-limitation (early-mid stage) shifting to P limitation (late stage) on upper slopes and persistent N-K co-limitation on lower slopes. (3) PCA identified F4 (1200 g/plant) and F1 (600 g/plant) as optimal for upper and lower slopes, respectively. This research provides a theoretical basis for precision fertilization in *Ochroma lagopus* plantations, emphasizing slope-specific nutrient status and element interactions for dosage optimization.

## 1. Introduction

Fertilization, as a key measure to promote the growth and development of trees, can effectively improve the soil nutrient status, supply a variety of essential nutrients for the growth and development of trees, and thus regulate the nutrient balance between trees and the forest. Water-soluble fertilizers have become an important tool for modern agriculture to improve quality and efficiency because of their high efficiency and precision [1]. Secondary macronutrients refer to essential mineral elements required by plants in moderate quantities, intermediate between macronutrients (N, P, K) and micronutrients (Fe, Mn, Zn, etc.). These principally include Ca, Mg, and S. In recent years, with the over-consumption of soil nutrients by intensive cultivation, the lack of secondary macronutrients has gradually become a key factor limiting crop yield and quality [2]. By directly supplementing Ca, Mg, S, and other elements, water-soluble fertilizers with trace elements have shown significant advantages in supplementing nutrition, preventing deficiency, and enhancing resistance [3]. Hilly ecosystems generally have significant slope differentiation [4], which profoundly affects the nutrient acquisition strategies of forest trees by reshaping soil and water transport and spatial allocation of nutrients. It has been shown that topographic gradients in hilly areas lead to significant spatial heterogeneity of soil secondary macronutrients [5]: upper slope positions are susceptible to persistent deficits of secondary macronutrients (e.g., Ca, Mg) in response to soil and water erosion due to strong surface runoff and shallow soils, which leads to a decrease in soil cation exchange and increased acidification [6], while the lower slope positions had a higher effectiveness of the middle elements due to organic matter deposition and clay enrichment, but the risk of secondary macronutrient antagonism and compound toxicity of S and heavy metals was significantly higher [7]. In addition, the amount of fertilizer applied as well as the time of fertilizer application determine the efficiency of fertilizer use by plants [8]. However, traditional fertilizer application mostly ignores topographic factors and uses homogenized doses, resulting in the double dilemma of insufficient fertilizer effectiveness on the upper slope positions and overloaded environmental loads on the lower slope positions, which makes it difficult to achieve a balance between economic and ecological benefits. Therefore, it is important to improve the efficiency of fertilizer use on different slopes for the sustainable development of agriculture and forestry.

Ecological chemometrics integrates multidisciplinary principles of biology, chemistry, and physics across multiple levels of organization; focuses on the ratio relationship of chemical elements in ecological processes; reveals the elemental coupling mechanism; and provides a comprehensive approach to explore ecosystem elemental interactions [9,10]. In plant nutrient metabolism, N, as a key component of enzymes, is widely involved in many important physiological activities, including photosynthesis, while P is closely related to cell growth, the division process, and protein synthesis, and K mediates osmoregulation and assimilate transport [11]. Studies have shown that secondary macronutrients may affect the N, P, and K contents of various organs through multiple pathways, with Ca promoting N uptake by roots by stabilizing the cell wall structure [12]; Mg, as a core chlorophyll constituent, is involved in photosynthesis in ATP synthesis, which in turn affects P assimilation [13,14]; and S, as a component of sulfur-containing amino acids, may indirectly affect the efficiency of N utilization through the regulation of protein synthesis [15]. In addition, the nutrient stoichiometric characteristics of tissues and organs in plants, such as N/P, N/K, and K/P, can reflect the efficiency of nutrient uptake and utilization, growth strategies, nutrient limitation conditions faced by plants, and their adaptive capacity to the environment, and are also crucial for elucidating the role of plants in the nutrient dynamics of ecosystems [16].

*Ochroma lagopus* (balsa tree), a large evergreen tree of the family Bombacaceae, is native to tropical low-altitude regions of the Americas that are free from typhoon disturbances. It is currently recognized as the lightest commercial timber species globally [17]. The species is characterized by rapid growth and a short production cycle [18] and can usually be harvested and utilized 5 to 6 years after planting. Its wood is light and strong [19] and has shown good application prospects in many fields such as aviation, navigation, industry, and civil use, and it is a high-quality material for the manufacture of ships, model airplanes, and paper making. However, *Ochroma lagopus*, as an emerging tree species in China, has not yet been well cultivated, especially in terms of nutrient management. Current cultivation practices are based on empirical over-fertilization strategies without scientific support. Current research on *Ochroma lagopus* focuses on wood properties [17], planting conditions [20,21], drought exercise studies [22], and N addition [23,24,25,26], whereas studies on precise fertilization of *Ochroma lagopus* at different slope positions have not been reported. Based on the phenomenon of slope-fertilization imbalance in *Ochroma lagopus* plantations, this study proposed the following scientific hypothesis: topography-driven differences in the effectiveness of secondary macronutrients dominate the nutrient partitioning strategy of *Ochroma lagopus* organs. In this paper, we investigated the nutrient allocation strategy of *Ochroma lagopus* organs by constructing a two-factor controlled trial of slope position-fertilization, aiming to provide a theoretical basis for the establishment of a precise fertilization system for *Ochroma lagopus* plantation forests based on the stand conditions and to help the high-quality development of the characteristic economic forest industry in tropical regions.

## 2. Materials and Methods

### 2.1. Study Area

The experimental site was located in the *Ochroma lagopus* plantation base of Mengwing Farm, Menglun Town, Mengla County, Xishuangbanna Dai Autonomous Prefecture, Yunnan Province (21°54′ N~21°55′ N, 101°10′ E~101°30′ E, elevation about 550 m). The experiment started on 15 May 2024 and ended on 15 December 2024. The site has a southwestern tropical monsoon climate with relatively obvious dry and wet seasons, with an average annual temperature of 21.9 °C, the lowest average monthly temperature in January (16.6 °C), and the highest in June (25.5 °C), an annual rainfall of 1520 mm, and precipitation mainly concentrated in May–October, which accounts for 85% of the annual precipitation. The soil is a typical red soil from Yunnan, classified as Acrisols according to the IUSS Working Group WRB (2015) standard [27], with a pH value of 4.28, organic matter of 23.6 g·kg^−1^, total nitrogen of 1.59 g·kg^−1^, total phosphorus of 0.32 g·kg^−1^, and total potassium of 12.0 g·kg^−1^.

### 2.2. Experimental Materials and Experimental Design

This study used 3-year-old *Ochroma lagopus* from Mengxing Farm, Menglun Town, Mengla County, Xishuangbanna, as research subjects. A water-soluble fertilizer experiment was conducted, with the fertilization rate determined based on the characteristics of the water-soluble fertilizer, local climatic conditions, production practices, and trials on fast-growing tropical tree species (including eucalyptus as a representative species) [28]. Combined with high soil moisture during the local rainy season, the fertilization method was direct application. The experiment was divided into an upper slope position (640 m above sea level) and a lower slope position (590 m above sea level), with the same slope direction (south slope) and similar slope gradient (about 40°), and 5 treatments were set up in each upper and lower slope position, totaling 10 treatments. Five gradients were set up in the upper and lower slope positions: CK (no water-soluble fertilizer treatment), F1 (water-soluble fertilizer 600 g/plant), F2 (water-soluble fertilizer 800 g/plant), F3 (water-soluble fertilizer 1000 g/plant), and F4 (water-soluble fertilizer 1200 g/plant) were set up in the upper slopes, and the water-soluble fertilizers were chosen as the secondary macronutrients water-soluble fertilizers (Mg ≥ 10%, S ≥ 10%, with the addition of Ca, Fe, Zn, B, and other trace elements) produced by Shandong Runkang Fertilizer Industry Co. (Dongying, China). Each treatment was 90 *Ochroma lagopus*, with 3 replications, totaling 900 *Ochroma lagopus* in 10 treatments. Fertilizer was applied directly according to the local climatic and production conditions, and the fertilizer was covered with soil after application. The first application of fertilizer started on 15 May 2024, and the last application was made on 15 June 2024 and the upper and lower slope positions were sampled at 120 days, 150, and 180 days after the end of fertilizer application, respectively.

### 2.3. Indicator Measurements

Tree height and diameter at breast height were measured once before the beginning of the experiment, and nine representative standard trees were selected for each treatment to measure their height and diameter at breast height at 120 days, 150, and 180 days after the end of fertilizer application, respectively. Tree height was measured with an altimeter (accurate to 0.1 cm), and breast diameter was measured with a breast diameter circumference ruler (accurate to 0.1 mm), and the data were recorded. Nine standard trees were randomly selected within each sample plot, and branches and leaves of the new shoots on the sunny side of the tree crown were collected with branch cutters, and the root system of the plant was dug. After labeling and cleaning, the samples were first treated in a forced-air oven at 120 °C for 30 min to rapidly inactivate enzymatic activity, thus preventing metabolite degradation. Subsequently, the temperature was reduced to 80 °C for drying until constant weight to completely remove moisture and avoid interference from residual water in chemical composition analysis. The dried samples were then ground using a mill, passed through a 100-mesh sieve, and stored in sealed plastic bags.

Total nitrogen (N) was determined by the Kjeldahl method (GB/T 7173-1987) [29]; organic nitrogen was converted to ammonium salts (NH_4_^+^) by digestion with concentrated sulfuric acid, ammonia (NH_3_) was liberated by adding alkali and absorbed in boric acid, then titrated with standard acid; total phosphorus (P) was measured by the vanadomolybdate yellow colorimetric method (NY/T 2421-2013) [30], orthophosphate reacts with ammonium molybdate-ammonium metavanadate in an acidic medium to form yellow phosphovanadomolybdate heteropoly acid, quantified colorimetrically at 440 nm; total potassium (K) was analyzed by flame photometry (GB/T 9836-1988) [30]; after digestion with HF-HClO_4_, potassium atoms were excited in a flame to emit characteristic spectral lines at 766.5 nm, with radiation intensity proportional to K^+^ concentration.

### 2.4. Statistical Analysis

Excel 2016 was used to pre-process the data, and SPSS 27.0 statistical analysis software was used to perform one-way ANOVA, significance analysis of variance, and correlation analysis on the experimental data, and the data were expressed as “mean ± standard deviation”; the plotting and principal component analysis were performed using Origin2024 software.

The data were standardized using SPSS 27.0 software to obtain new data and passed the test of applicability of data correlation matrix factor analysis. Y1 = Z1 × X1 + Z2 × X2 + …… + Z20 × X20, Y2 = Z1 × X1 + Z2 × X2 + …… + Z20 × X20. Y = Y1 × Principal Component 1 Variance Contribution + Y2 × Principal Component 2 Variance Contribution. Y1 is the principal component 1 score, Y2 is the principal component 2 score, Y is the composite score, Z is the coefficient of the principal component scores, and X is the standardized value of growth and physiological indexes.

## 3. Results

### 3.1. Effects of Fertilizer Application on Tree Height and Diameter at Breast Height of Ochroma lagopus Plantations on Different Slope Positions

**Figure 1:**
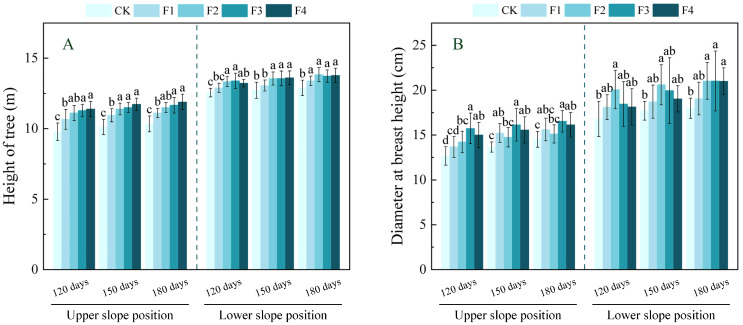
(**A**,**B**) Effect of fertilization on tree height and diameter at breast height (DBH) of *Ochroma lagopus* at different slopes. Note: different lowercase letters indicate significant differences between different fertilization treatments at the same sampling time (*p* < 0.05). CK refers to no water-soluble fertilizer treatment, 600 g/water-soluble fertilizer applied to F1, 800 g/water-soluble fertilizer applied to F2, 1000 g/water-soluble fertilizer applied to F3, and 1200 g/water-soluble fertilizer applied to F4.

As shown in Figure 1, the tree height of *Ochroma lagopus* trees at the upper slope position increased with fertilizer dosage. At 180 days, tree height under all fertilizer treatments was significantly higher than CK, reaching its maximum in the F4 treatment with a 15.1% increase compared to CK at 180 days (*p* < 0.05). Diameter at breast height (DBH) showed variable responses, peaking in the F3 treatment at 180 days with a significant increase of 13.8% versus CK at 180 days (*p* < 0.05).

At the lower slope position, both tree height and DBH exhibited an initial increase followed by a decrease across fertilization timings. By 180 days, both parameters peaked in the F2 treatment, showing significant increases of 7.3% (height) and 16.9% (DBH) relative to CK at 180 days (*p* < 0.05).

### 3.2. Effect of Fertilization on Leaf Nitrogen, Phosphorus, and Potassium and Their Stoichiometric Characteristics of Ochroma lagopus at Different Slope Positions

**Figure 2:**
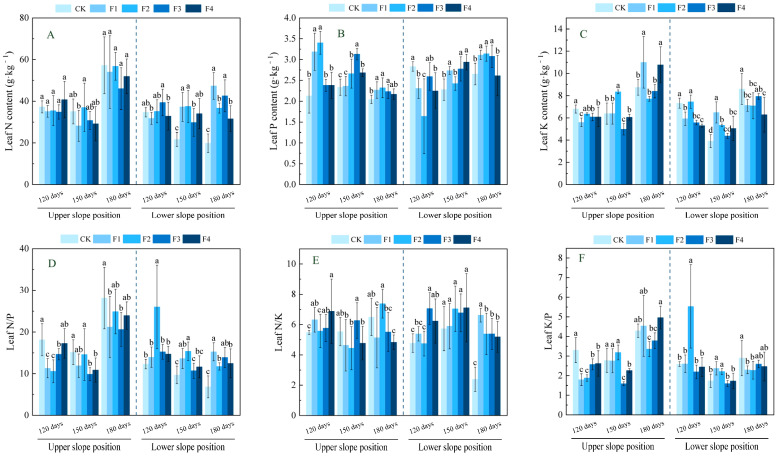
Effects of fertilization on nitrogen, phosphorus, and potassium, and their stoichiometric characteristics in the leaves of *Ochroma lagopus* at different slopes. Note: different lowercase letters indicate significant differences between different fertilization treatments at the same sampling time (*p* < 0.05). Figure (**A**) refers to leaf N content, Figure (**B**) refers to leaf P content, Figure (**C**) refers to leaf K content, Figure (**D**) refers to leaf N/P, Figure (**E**) refers to leaf N/K, and Figure (**F**) refers to leaf K/P.

As can be seen from Figure 2, with the increase in fertilizer application, the nutrient characteristics of *Ochroma lagopus* leaves at upper and lower slope positions showed different performance under different fertilizer application times. At the upper slope position, leaf N content showed no significant changes across fertilization treatments at different fertilization times compared to the control. Leaf P content reached its maximum values at different fertilization times in the F2, F4, and F2 treatments, showing significant increases of 60.0%, 34.0%, and 10.1%, respectively, compared to CK in each time period (*p* < 0.05). Leaf K content at 120 days in the F2 treatment decreased significantly by 17.6% compared to CK (*p* < 0.05), while at 150 and 180 days, it reached its maximum values in the F2 and F1 treatments, respectively, showing significant increases of 30.7% and 25.7% compared to CK (*p* < 0.05). The leaf N/P ratio generally showed a downward trend across different fertilization times, with the F2 treatment at 120 days decreasing significantly by 41.8% compared to CK (*p* < 0.05), and by 150 and 180 days, the F3 treatment had the lowest values, decreasing significantly by 34.9% and 26.8%, respectively, compared to CK (*p* < 0.05). The leaf N/K ratio at 120 days reached its maximum value in the F4 treatment, increasing significantly by 25.5% compared to CK (*p* < 0.05), while by 150 and 180 days, the lowest values were observed in the F2 and F4 treatments, respectively, decreasing significantly by 19.8% and 25.6% compared to CK (*p* < 0.05). The leaf K/P ratio at 120 days in the F1 treatment decreased significantly by 45.7% compared to CK (*p* < 0.05), and by 150 and 180 days, the lowest values were observed in the F3 and F2 treatments, respectively, decreasing significantly by 42.8% and 21.8% compared to CK (*p* < 0.05).

At the lower slope position, leaf N content reached its maximum value in the F2 treatment at 150 days after fertilization and in the F1 treatment at 180 days, showing significant increases of 73.0% and 138.9%, respectively, compared to CK (*p* < 0.05), while showing no significant change at 120 days. Leaf P content at 120 days in the F2 treatment decreased significantly by 42.2% compared to CK (*p* < 0.05), while by 150 and 180 days, it reached its maximum value in the F4 and F2 treatments, respectively, showing significant increases of 29.0% and 18.5% compared to CK (*p* < 0.05). Leaf K content at 120 and 180 days in the F4 treatment decreased significantly by 27.7% and 27.0%, respectively, compared to CK (*p* < 0.05), while at 150 days, it increased significantly by 66.3% in the F1 treatment compared to CK (*p* < 0.05). The leaf N/P ratio reached its maximum value in the F2 treatment at 120 and 150 days, showing significant increases of 111.6% and 58.8%, respectively, compared to CK (*p* < 0.05), and by 180 days, it increased significantly by 123.1% in the F1 treatment compared to CK (*p* < 0.05). The leaf N/K ratio reached its maximum value in the F1 treatment at 120 days and in the F3 treatment at 180 days, showing significant increases of 47.8% and 177.0%, respectively, compared to CK (*p* < 0.05), while showing no significant change at 150 days; the leaf K/P ratio reached its maximum value in the F2 treatment at 120 days and in the F1 treatment at 150 days, showing significant increases of 113.9% and 36.7%, respectively, compared to CK (*p* < 0.05), and by 180 days, it decreased significantly by 21.5% in the F2 treatment compared to CK (*p* < 0.05).

### 3.3. Effect of Fertilization on Nitrogen, Phosphorus, and Potassium and Their Stoichiometric Characteristics of Ochroma lagopus Branches at Different Slope Positions

**Figure 3:**
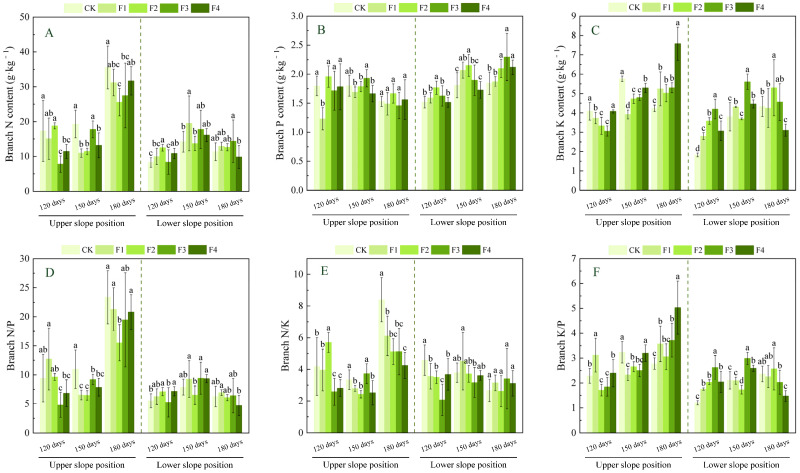
Effects of fertilization on nitrogen, phosphorus, and potassium and their stoichiometric characteristics in the branches of *Ochroma lagopus* at different slopes. Note: different lowercase letters indicate significant differences between different fertilization treatments at the same sampling time (*p* < 0.05). Figure (**A**) refers to branch N content, Figure (**B**) refers to branch P content, Figure (**C**) refers to branch K content, Figure (**D**) refers to branch N/P, Figure (**E**) refers to branch N/K, and Figure (**F**) refers to branch K/P.

As shown in Figure 3, with increasing fertilization rate, branch N content at the upper slope position reached its minimum value in the F3 treatment at 120 days after fertilization, the F1 treatment at 150 days, and the F2 treatment at 180 days, showing significant decreases of 55.0%, 43.2%, and 28.1%, respectively, compared to CK (*p* < 0.05); branch P content showed a significant decrease only at 120 days, with the F1 treatment decreasing by 31.4% compared to CK (*p* < 0.05), while at 150 and 180 days, there were no significant changes across fertilization treatments. Branch K content reached its minimum value in the F3 treatment at 120 days and in the F1 treatment at 150 days, showing significant decreases of 25.0% and 32.0%, respectively, compared to CK (*p* < 0.05), while by 180 days, it increased significantly by 78.8% in the F4 treatment compared to CK (*p* < 0.05). The branch N/P ratio at 120 days in the F3 treatment decreased significantly by 48.8% compared to CK (*p* < 0.05), while by 150 and 180 days, the F2 treatment had the lowest values, decreasing significantly by 41.3% and 33.5%, respectively, compared to CK (*p* < 0.05); the branch N/K ratio at 120 days reached its maximum value in the F2 treatment, increasing significantly by 36.5% compared to CK (*p* < 0.05), while by 150 and 180 days, the lowest values were observed in the F2 and F4 treatments, respectively, decreasing significantly by 27.3% and 49.4% compared to CK (*p* < 0.05). The branch K/P ratio reached its maximum value in the F1 treatment at 120 days and in the F4 treatment at 180 days, showing significant increases of 36.5% and 81.1%, respectively, compared to CK (*p* < 0.05), while at 150 days, it decreased significantly by 28.0% in the F1 treatment compared to CK (*p* < 0.05).

At the lower slope position, branch N content reached its maximum value under the F2 treatment at 120 days after fertilization and under the F1 treatment at 150 days, showing significant increases of 50.7% and 36.9%, respectively, compared to CK (*p* < 0.05), while no significant changes were observed among fertilization treatments at 180 days. Both branch P and branch K contents exhibited a trend of initially increasing and then decreasing across all fertilization times, with branch P content peaking under the F2 treatment at both 120 and 150 days but reaching its maximum under the F3 treatment at 180 days, resulting in significant increases of 16.4%, 18.8%, and 24.6%, respectively, compared to CK (*p* < 0.05). Branch K content peaked under the F3 treatment at 120 and 150 days but reached its maximum under the F2 treatment at 180 days, showing significant increases of 130.3%, 47.7%, and 22.2%, respectively, compared to CK (*p* < 0.05). The branch N/P ratio reached its maximum under the F2 treatment at 120 days, showing a significant increase of 29.7% compared to CK (*p* < 0.05), but showed no significant changes at 150 and 180 days; the branch N/K ratio was lowest under the F3 treatment at 120 days, showing a significant decrease of 54.8% compared to CK (*p* < 0.05), with no significant changes at 150 and 180 days. The branch K/P ratio peaked under the F3 treatment at 120 and 150 days, showing significant increases of 117.5% and 41.9%, respectively, compared to CK (*p* < 0.05), but exhibited a decreasing trend by 180 days, with the F4 treatment showing a significant decrease of 37.6% compared to CK (*p* < 0.05).

### 3.4. Effect of Fertilization on Nitrogen, Phosphorus, and Potassium and Their Stoichiometric Characteristics in Roots of Ochroma lagopus at Different Slope Positions

**Figure 4:**
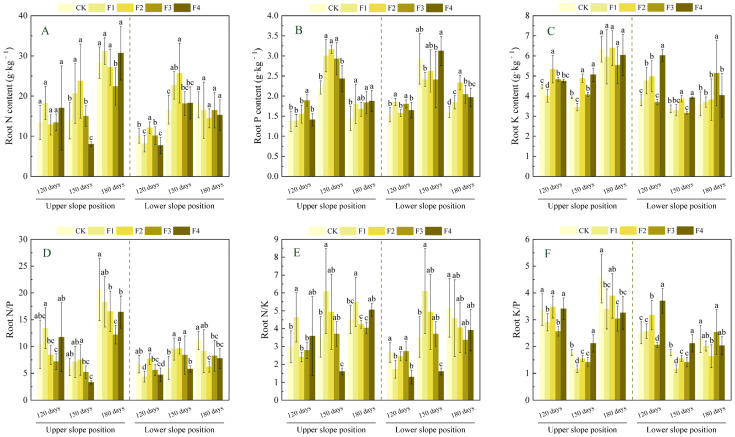
Effects of fertilization on nitrogen, phosphorus, and potassium and their stoichiometric characteristics in the roots of *Ochroma lagopus* at different slopes. Note: different lowercase letters indicate significant differences between different fertilization treatments at the same sampling time (*p* < 0.05). Figure (**A**) refers to root N content, Figure (**B**) refers to root P content, Figure (**C**) refers to root K content, Figure (**D**) refers to root N/P, Figure (**E**) refers to root N/K, and Figure (**F**) refers to root K/P.

As shown in Figure 4, with increasing fertilizer application rate, the root N content at the upper slope position reached its maximum under the F2 treatment at 150 days after fertilization, showing a significant increase of 71.3% compared to CK (*p* < 0.05), while it was lowest under the F3 treatment at 180 days, showing a significant decrease of 20.9% (*p* < 0.05), with no significant changes observed at 120 days. The root P content peaked under the F3, F2, and F4 treatments at different fertilization times, showing significant increases of 38.3%, 42.8%, and 29.9%, respectively, compared to CK (*p* < 0.05). The root K content reached its maximum under the F2 treatment at 120 days and the F4 treatment at 150 days, showing significant increases of 19.2% and 29.4%, respectively, compared to CK (*p* < 0.05), while no significant changes were observed among fertilization treatments at 180 days. The root N/P ratio was lowest under the F4 treatment at 150 days and the F3 treatment at 180 days, showing significant decreases of 46.0% and 40.9%, respectively, compared to CK (*p* < 0.05), with no significant changes at 120 days. The root N/K ratio reached its maximum under the F1 treatment at all fertilization times, showing significant increases of 55.4%, 72.4%, and 21.8%, respectively, compared to CK (*p* < 0.05). The root K/P ratio was lowest under the F3 treatment at 120 and 180 days, showing significant decreases of 23.9% and 33.0%, respectively, compared to CK (*p* < 0.05), while at 150 days, the F1 treatment showed a significant decrease of 34.5% compared to CK (*p* < 0.05).

At the lower slope position, root N content reached its maximum under the F2 treatment at 120 and 150 days after fertilization, showing significant increases of 20.3% and 54.9%, respectively, compared to CK (*p* < 0.05), while no significant changes were observed among treatments at 180 days. The root P content peaked under the F1 treatment at 120 days and the F2 treatment at 180 days, showing significant increases of 20.8% and 45.6%, respectively, compared to CK (*p* < 0.05), with no significant changes at 150 days. The root K content reached its maximum under the F4 treatment at 120 and 150 days and under the F3 treatment at 180 days, showing significant increases of 58.8%, 16.5%, and 42.5%, respectively, compared to CK (*p* < 0.05). The root N/P ratio was lowest under the F1 treatment at 120 days and the F2 treatment at 180 days, showing significant decreases of 34.7% and 44.1%, respectively, compared to CK (*p* < 0.05), while it reached its maximum under the F2 treatment at 150 days, showing a significant increase of 60.4% (*p* < 0.05). The root N/K ratio was lowest under the F4 treatment at 120 days, showing a significant decrease of 51.7% compared to CK (*p* < 0.05); reached its maximum under the F1 treatment at 150 days, showing a significant increase of 72.4% (*p* < 0.05); and showed no significant changes at 180 days. The root K/P ratio peaked under the F4 treatment at 120 and 150 days, showing significant increases of 47.4% and 19.3%, respectively, compared to CK (*p* < 0.05), while it was lowest under the F2 treatment at 180 days, showing a significant decrease of 28.3% compared to CK (*p* < 0.05).

### 3.5. Correlation Analysis

**Figure 5:**
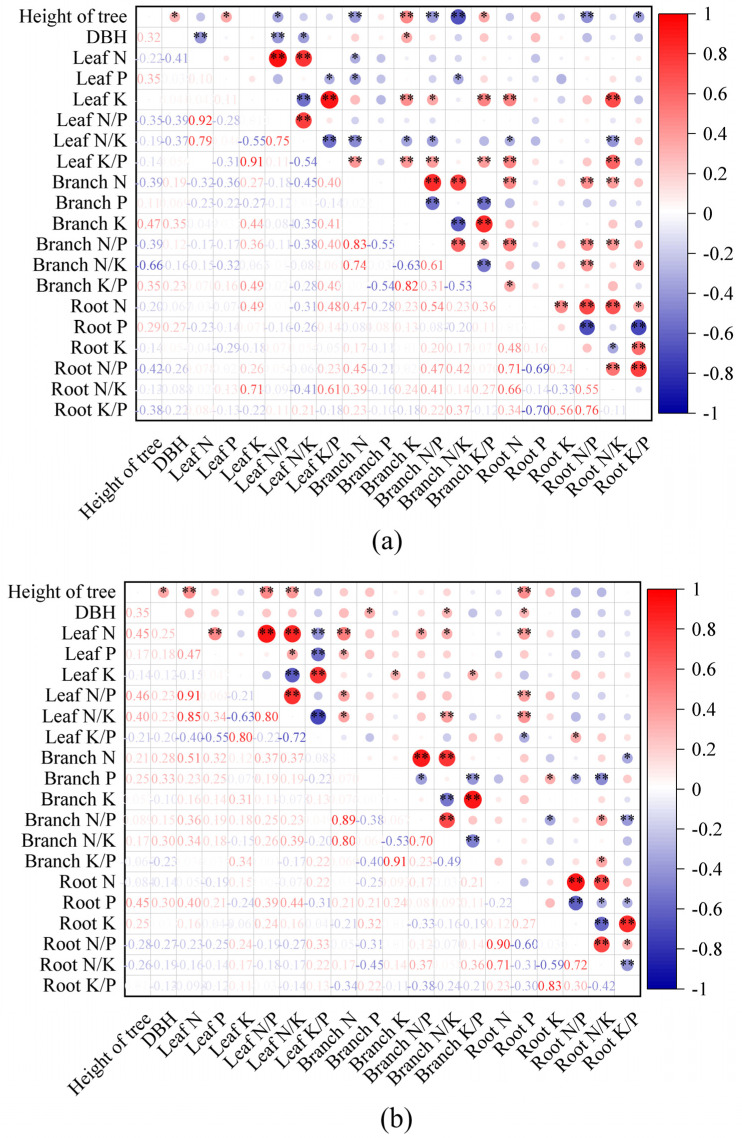
Correlation analysis of each index of *Ochroma lagopus* in different slope positions under sampling detection for 180 days after fertilization. Note: * indicates *p* < 0.05, ** indicates *p* < 0.01. Figure (**a**) refers to the upper slope position and Figure (**b**) refers to the lower slope position.

As can be seen from Figure 5, there was a general correlation between the growth indexes of *Ochroma lagopus* and the nutrient indexes under the 180 days sampling test of fertilizer application. As can be seen from Figure 5a, in the upper slope position, tree height was highly significantly positively correlated with branch K (*p* < 0.01); highly significantly negatively correlated with branch N, branch N/P, branch N/K, and root N/P (*p* < 0.01); highly significantly positively correlated with DBH, leaf P, and branch K/P (*p* < 0.05); and significantly negatively correlated with leaf N/P and root K/P (*p* < 0.05). DBH was highly significantly negatively correlated with leaf N and leaf N/P (*p* < 0.01), significantly positively correlated with root K (*p* < 0.05), and significantly negatively correlated with leaf N/K (*p* < 0.05). Leaf N was significantly positively correlated with leaf N/P and leaf N/P (*p* < 0.01) and significantly negatively correlated with branch N (*p* < 0.05). Leaf P was significantly negatively correlated (*p* < 0.05) with leaf K/P, branch N, and branch N/K. Leaf K was significantly positively correlated with leaf K/P, branch K, branch K/P, root N, and root N/K (*p* < 0.01), significantly negatively correlated with leaf N/K (*p* < 0.01), and significantly positively correlated with branch N/P (*p* < 0.05).

As can be seen in Figure 5b, in the downslope position, tree height was highly significantly and positively correlated with leaf N, leaf N/P, leaf N/K, and root P (*p* < 0.01) and significantly and positively correlated with DBH (*p* < 0.05). DBH was significantly positively correlated with branch P, branch N/K, and root P (*p* < 0.05). Leaf N was significantly positively correlated (*p* < 0.01) with leaf P, leaf N/P, leaf N/K, branch N, and root P, significantly negatively correlated (*p* < 0.01) with leaf K/P, and significantly positively correlated (*p* < 0.05) with branch N/P and branch N/K. Leaf P was highly significantly negatively correlated with leaf K/P (*p* < 0.01) and significantly positively correlated with leaf N/K and branch N (*p* < 0.05). Leaf K was highly significantly and positively correlated with leaf K/P (*p* < 0.01), highly significantly and negatively correlated with leaf N/K (*p* < 0.01), and significantly and positively correlated with branch K and branch K/P (*p* < 0.05).

### 3.6. Principal Component Analysis (PCA)

**Figure 6:**
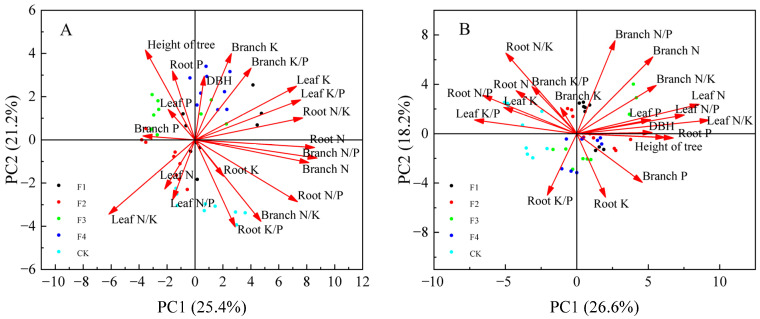
Principal component analysis of each index of *Ochroma lagopus* in different slope positions under 180 days of sampling detection for 180 days after fertilization. Note: Figure (**A**) refers to the upper slope position and Figure (**B**) refers to the lower slope position.

Principal component analysis was performed for each indicator of *Ochroma lagopus* at different slope positions at 180 days of fertilizer application (Figure 6). The cumulative variance contributions of the first two principal components of light wood in the upper slope position and lower slope position at 180 days of fertilizer application were 46.6% and 44.8%, so the first two principal components were able to effectively explain the characteristics of the response to fertilizer treatment for light wood in different slope positions. As shown in Figure 6A, the weight coefficients of branch N/P, root N, and branch N were larger in PC1, and the weight coefficients of tree height and branch K were larger in PC2; and as shown in Figure 6B, the weight coefficients of leaf N/K and leaf N were larger in PC1, and the weight coefficients of branch N/P, branch N, and root N/K were larger in PC2. In summary, leaf N, leaf N/K, branch N, branch N/P, and root N basically responded to the adaptive characteristics of *Ochroma lagopus* on different slopes to fertilization.

As can be seen from Table 1 and Table 2, the most favorable fertilization levels for the growth of *Ochroma lagopus* at different slope positions can be derived from the principal component analysis. With the increase in fertilization, the upper slope position of *Ochroma lagopus* scored the highest in F4 fertilization treatment, followed by F1 fertilization treatment, and CK treatment scored the lowest; the lower slope position of *Ochroma lagopus* scored the highest in F1 fertilization treatment, followed by F3 fertilization treatment, and CK treatment scored the lowest. In summary, it can be seen that reasonable fertilization is beneficial to the growth of *Ochroma lagopus* in different slope positions, while CK without fertilization treatment is not beneficial to the growth of *Ochroma lagopus*.

## 4. Discussion

### 4.1. Effects of Fertilizer Application on the Growth of Ochroma lagopus Plantations on Different Slope Positions

The growth of tree height and DBH can be visualized in the growth of forest trees [31]. As essential elements for plant growth and development, the application of water-soluble fertilizers with moderate elements can make the nutrient absorption of plants more balanced, and some studies have shown that the application of a certain amount of water-soluble fertilizers with moderate elements can promote the growth of plants and increase the yield of crops [32]. In this study, it was found that fertilization could promote the growth of *Ochroma lagopus*, and the height of trees in both upper and lower slope positions showed an increasing trend with the increase in fertilizer application, which is similar to the findings of Kulmann et al. [33] regarding the significant effects of fertilization and stand uniformity on the growth of Brazilian *Pinus taeda* plantations. This dose-dependent response is likely attributable to the synergistic enhancement of photosynthetic capacity and nutrient uptake mediated by secondary elements. DBH, on the other hand, showed a tendency to increase and then decrease, with the upper and lower slope positions having the largest DBH under F3 and F2 treatments, respectively. This aligns with the inference by Lenin et al. [34] that in the nutrient-poor upper slope position, the F3 treatment enhances xylem development via Ca supplementation, yet higher doses trigger K^+^ uptake antagonism, constraining radial growth; the lower slope position can meet the demand by F2 treatment due to soil nutrient enrichment, and excessive fertilization leads to S^2−^ accumulation interfering with K^+^ metabolism, instead of inhibiting DBH expansion. This indicates that fertilization strategies should consider slope position differences: nutrient-deficient slope positions may moderately increase dosage to compensate for nutrient deficiency, while nutrient-rich slope positions need to control dosage to avoid nutrient element antagonism. This is similar to the findings of Ou and Kang [35] regarding the slope position effects on annual growth dynamics and growth rhythms of *Manglietia yuyuanensis* saplings.

### 4.2. Effect of Fertilizer Application on N, P, and K Contents and Their Stoichiometric Characteristics of Various Organs of Ochroma lagopus at Different Slope Positions

The nutrient content of plant organs can characterize the nutrient uptake status of plants and their adaptability to the environment to a certain extent. N, P, and K are essential bulk elements for plant growth [36]; their accumulation and organ distribution directly affect plant growth. N and P are essential building blocks of genetic material and biological proteins [37], while K regulates ion homeostasis and cellular expansion [38] and plays an important role in plants. In this study, we found that the upper slope position with poor soil significantly elevated leaf P content with increasing fertilizer application compared with CK, which might be related to the promotion of organic phosphorus mineralization by Mg^2+^ through activation of phosphatase [39], thus enhancing the efficiency of P uptake by leaves. In contrast, the initial decline (17.6% in the F2 treatment at 120 days) and late rebound (25.7% increase in the F1 treatment at 180 days) in leaf K content implied that low doses of Ca^2+^ alleviated K^+^ loss, whereas high doses inhibited its uptake. Branches N and K contents were significantly decreased under medium- to high-level fertilization F2–F3 treatments, probably because excess Ca^2+^ led to cation competition (e.g., Ca^2+^ and K^+^) and limited nutrient availability [40], inhibiting the branches’ access to N, and K, whereas root P and K were elevated in the F2–F4 treatment, suggesting that Ca^2+^ expanded the surface area for P and K uptake by enhancing root cell wall stability and extending root longevity [41], while S was involved in root thionin synthesis, possibly enhancing phosphorus transport protein activity. Lower slope position due to soil nutrient enrichment: leaf N content increased significantly by 138.9% in the F1 treatment at 180 days in the late fertilization period, probably because of the optimization of the soil nutrient structure by the medium elements, which promoted the release of N conversion and improved the efficiency of plant N use; while branch P and branch K content showed a tendency to rise and then fall, which may be related to the nutrient redistribution in the body of the plant and physiological metabolism in the stage of the changes, Mg^2+^ promotes the accumulation of photosynthetic products in newborn branches and drives the allocation of P and K to branches. The persistent accumulation of P and K content in roots (45.6% increase in root P content and 58.8% increase in root K content) suggests that *Ochroma lagopus* buffers metabolic fluctuations in the upper part of the ground under eutrophic conditions through the “energy storage” strategy of the root system.

N/P, N/K, and K/P are often used as important indicators for assessing the nutrient supply status of plant growth. Specifically, Yan et al. [42] established threshold criteria: N/P > 16 indicates phosphorus (P) limitation, whereas N/P < 14 suggests nitrogen (N) limitation. Additionally, Wright et al. [43] proposed that N/K > 2.1 combined with K/P < 3.4 reflects potassium (K) limitation. These indicators are useful for exploring plant growth and development. In this study, we found that the N/P of each organ in the upper slope position was generally greater than 16 in each fertilization treatment at 180 days of fertilization, which was greater than the national average of plant N/P (14.4) [44], indicating that P was the main limiting element in the late stage of fertilization, dominating canopy photosynthesis and below-ground resource capturing. In contrast, branches and roots had N/P < 14 at 120 and 150 days of fertilization and showed a decreasing trend with increasing fertilization, indicating that plants preferentially allocated limited N resources to leaves to maintain photosynthetic capacity, which is similar to the research results of Wen et al. [25] regarding the effects of nitrogen addition on the growth of *Ochroma lagopus* plantations. The resultant relative deprivation of branch and root N content may be due to the inhibition of ammonium root ion NH_4_^+^ uptake by high doses of Ca^2+^ in infertile stands. At the same time, N/K > 2.1 for each organ and K/P < 3.4 at 120 and 150 days in the pre-intermediate stage of fertilization indicated the existence of K limitation, which might originate from Ca^2+^/K^+^ ion channel competition, and it is worth noting that K/P > 3.4 at 180 days in the late stage of fertilization indicated that the water-soluble fertilizers with a medium amount of elements were able to alleviate K limitation. *Ochroma lagopus* in the lower slope position had whole-organ N/P < 14 and N/K > 2.1, combined with K/P < 3.4, suggesting that its growth was co-limited by N-K, which was attributed to the metabolic imbalance triggered by the excess of S^2−^ in the eutrophic soil, which could promote N uptake in a certain range, but the excess of S^2−^ was unfavorable to N uptake [45], resulting in lower N/P, while S^2−^ inhibits the phloem loading efficiency of K^+^ and exacerbates K limitation.

### 4.3. Analysis of the Relationship Between Fertilization and the Growth of Ochroma lagopus and N, P, and K Contents of Various Organs and Their Stoichiometric Characteristics at Different Slope Positions

In this study, tree height in the upper slope position was highly significantly and positively correlated with leaf N, leaf N/P, leaf N/K, and root P (*p* < 0.01), and DBH was significantly and positively correlated with root K (*p* < 0.05); in the lower slope position, tree height was highly significantly and positively correlated with leaf N, leaf N/P, leaf N/K, and root P (*p* < 0.01), and DBH was significantly and positively correlated with branch P, branch N/K, and root P (*p* < 0.05). It showed that the growth process of *Ochroma lagopus* was mainly affected by the N and P contents in each organ significantly and that the growth of tree height and DBH could be effectively promoted by adjusting the nutrient content ratio of each organ. Principal component analysis showed that branch N/P, root N, and branch N loaded more on the principal components for upper slope position lagopus (Figure 6A), and leaf N/K and leaf N loaded more on the principal components for lower slope position lagopus (Figure 6B), and that upper slope position lagopus scored the highest in F4 fertilization treatment, and lower slope position lagopus scored the highest in F1 fertilization treatment (Table 1). Combining the results of correlation and principal component analysis showed that the upper slope position of *Ochroma lagopus* grew better under F4 fertilization treatment due to poor soil, and the lower slope position grew better under F1 treatment due to nutrient enrichment. In addition, the N and P contents of each organ and their stoichiometric characteristics could better respond to the adaptation of *Ochroma lagopus* to the application of water-soluble fertilizers with moderate elements.

## 5. Conclusions

Nutrient allocation strategies in *Ochroma lagopus* organs were primarily governed by terrain-driven disparities in secondary element availability. The experimental results showed that water-soluble fertilizers with moderate elements could synergistically enhance photosynthesis and promote the growth of *Ochroma lagopus* tree height, but over-fertilization would inhibit the radial growth of DBH due to ionic antagonism (such as S^2−^ and K^+^). Differences in soil nutrient conditions at different slope positions significantly affected the nutrient use strategy of *Ochroma lagopus*: the upper slope position had poor soil, and fertilization significantly increased leaf P content, but medium- and high-dose fertilization led to a decrease in branch N and K content and an increase in root P and K content; the lower slope position had rich soil, with a significant increase in leaf N content during the later stages of fertilization, and the root system achieved “energy storage” by accumulating P and K. The stoichiometric threshold diagnosis further clarified that the upper slope position was N-K co-restricted in the first and middle stages and then shifted to P-restricted in the later stages, while the lower slope position was in N-K co-restricted status for the whole period. Based on the principal component analysis, 1200 g/plant (F4) in the upper slope position and 600 g/plant (F1) in the lower slope position were identified as the optimal fertilization treatments. The results of this research provide a scientific basis for precise fertilization management of *Ochroma lagopus* plantation forests. In actual production, it is necessary to optimize the fertilization dose by combining the nutrient status of the soil and elemental interactions at the slope position in order to achieve a balance between the growth promotion of *Ochroma lagopus* and the efficient use of nutrients and to provide a reference for the fertilization management of other tropical fast-growing tree species under complex terrain conditions. However, to more precisely optimize fertilization strategies, future research needs to further investigate (1) the long-term dynamic variation patterns of soil nutrients at different slope positions (especially key limiting factors) and their driving mechanisms; (2) the more complex interaction mechanisms between major nutrient elements (such as N, P, and K) and other micronutrients and their comprehensive effects on balsa wood growth; and (3) the evaluation of fertilization effects based on long-term fixed-position observations to validate and refine existing models.

## Figures and Tables

**Table 1:** Comprehensive evaluation of fertilization on *Ochroma lagopus* at different slopes under a 180-day sampling time.

Slope Position	Fertilization	Y1	Y2	Y (Composite Scores (Y))	Ranking
Upper slope position	CK	0.040	−1.614	−0.332	5
	F1	0.999	−0.329	0.184	2
	F2	−1.225	0.720	−0.158	3
	F3	−1.018	0.044	−0.249	4
	F4	1.204	1.179	0.556	1
Lower slope position	CK	−0.287	−1.475	−0.345	5
	F1	0.974	1.072	0.454	1
	F2	−0.542	0.313	−0.087	3
	F3	1.073	−0.504	0.193	2
	F4	−1.218	0.595	−0.216	4

Note: Y1 is the principal component 1 score, Y2 is the principal component 2 score, and Y is the comprehensive evaluation score.

**Table 2:** Principal component score matrix of *Ochroma lagopus* at different slope positions under 180 days of sampling detection time.

Index	Upper Slope Position		Lower Slope Position	
	Y1	Y2	Y1	Y2
Height of tree	−0.050	0.226	−0.023	0.009
DBH	−0.044	0.063	−0.091	−0.134
Leaf N	0.049	0.028	0.170	0.145
Leaf P	−0.102	0.073	0.037	−0.034
Leaf K	0.245	0.019	0.187	−0.364
Leaf N/P	0.084	−0.009	0.152	0.180
Leaf N/K	−0.104	0.009	0.056	0.303
Leaf K/P	0.280	−0.012	0.137	−0.284
Branch N	0.084	−0.168	0.301	−0.099
Branch P	0.093	0.051	−0.103	−0.128
Branch K	0.125	0.285	−0.042	−0.022
Branch N/P	0.016	−0.167	0.321	−0.055
Branch N/K	−0.006	−0.303	0.263	−0.048
Branch K/P	0.044	0.211	0.001	0.021
Root N	0.110	0.058	−0.051	0.066
Root P	0.011	−0.051	−0.043	0.067
Root K	−0.113	0.090	0.020	0.016
Root N/P	0.067	0.036	−0.014	0.018
Root N/K	0.227	−0.028	−0.050	0.051
Root K/P	−0.092	0.078	0.035	−0.046

Note: DBH refers to diameter at breast height; Leaf N, P, K refers to leaf N, P, and K concentrations; Leaf N/P, N/K, K/P refers to leaf stoichiometric ratios; Branch N, P, K refers to branch N, P, and K concentrations; Branch N/P, N/K, K/P refers to branch stoichiometric ratios; Root N, P, K refers to root N, P, and K concentrations; and Root N/P, N/K, K/P refers to root stoichiometric ratios.

## Data Availability

Data will be made available on request.
